# Pilot study of a basic individualized cognitive behavioral therapy program for chronic pain in Japan

**DOI:** 10.1186/s13030-020-00176-w

**Published:** 2020-03-10

**Authors:** Hiroki Hosogoshi, Kazunori Iwasa, Takaki Fukumori, Yuriko Takagishi, Yoshitake Takebayashi, Tomonori Adachi, Yuki Oe, Yukino Tairako, Yumiko Takao, Hiroyuki Nishie, Ayako Kanie, Masaki Kitahara, Kiyoka Enomoto, Hirono Ishii, Issei Shinmei, Masaru Horikoshi, Masahiko Shibata

**Affiliations:** 1 Department of Sociology, Faculty of Sociology, Kansai University, 3-3-35 Yamate-cho, Suita-shi, Osaka, 564-8680 Japan; 2 National Center for Cognitive Behavior Therapy and Research, National Center of Neurology and Psychiatry, 4-1-1 Ogawahigashi-cho, Kodaira-shi, Tokyo, 187-8551 Japan; 3 Faculty of Education, Shujitsu University, 1-6-1 Nishigawara, Naka-ku, Okayama-shi, Okayama, 703-8516 Japan; 4 Graduate School of Integrated Arts and Sciences, Tokushima University, 1-1 Minamijosanjima-cho, Tokushima-shi, Tokushima, 770-8502 Japan; 5 Department of Psychology, Surugadai University, 698 Azu, Hanno-shi, Saitama, 357-8555 Japan; 6 Department of Health Risk Communication, School of Medicine, Fukushima Medical University, 1 Hikarigaoka, Fukushima-shi, Fukushima, 960-1295 Japan; 7 Pain Management Clinic, Shiga University of Medical Science Hospital, Seta Tsukinowa-cho, Otsu-shi, Shiga 520-2192 Japan; 8 Department of Neuropsychiatry, Kyorin University School of Medicine, 6-20-2 Shinkawa, Mitaka-shi, Tokyo, 181-8611 Japan; 9 Department of Psychology, Meiji Gakuin University, 1-2-37 Shirokanedai, Minato-ku, Tokyo, 108-8636 Japan; 10 Department of Pain Medicine, Hyogo College of Medicine, 1-1 Mukogawa-cho, Nishinomiya-shi, Hyogo 663-8501 Japan; 11 Anesthesiology and Intensive Care 2, Kawasaki Medical School, 577 Matsushima, Kurashiki-shi, Okayama, 701-0192 Japan; 12 Department of Anesthesiology, Yokohama City University Medical Center, 4-57 Urafune-cho, Minami-ku, Yokohama-shi, Kanagawa 232-0024 Japan; 13 Graduate School of Human Sciences, Osaka University, 1-2 Yamadaoka, Suita-shi, Osaka, 565-0871 Japan; 14 Counseling Office, Japan Women’s University, 1-1-1 Nishiikuta, Tama-ku, Kawasaki-shi, Kanagawa 214-8565 Japan; 15 Department of Neurology, National Center of Neurology and Psychiatry, 4-1-1 Ogawahigashi-cho, Kodaira-shi, Tokyo, 187-8553 Japan; 16 TCBT Counseling Office, Cosmos Kichijoji Building 2F, 2-8-4 Kichijojihoncho, Musashino-shi, Tokyo, 180-0004 Japan; 17 Department of Health Science, Naragakuen University, 3-15-1 Nakatomigaoka, Nara-shi, Nara, 631-8524 Japan

**Keywords:** Chronic pain, Cognitive behavioral therapy, Psychotherapy, Multidisciplinary treatment, Quality of life (QOL), Japanese, Asia, Feasibility

## Abstract

**Background:**

Chronic pain is a major health problem, and cognitive behavioral therapy (CBT) is its recommended treatment; however, efforts to develop CBT programs for chronic pain and assess their feasibility are remarkably delayed in Asia. Therefore, we conducted this pilot study to develop a basic individualized CBT for chronic pain (CBT-CP) and assessed its feasibility for use in Japan.

**Methods:**

Our study was an open-labeled before–after trial without a control group conducted cooperatively in five Japanese tertiary care hospitals. Of 24 outpatients, 15, age 20–80, who experienced chronic pain for at least three months were eligible. They underwent an eight-session CBT-CP consisting of relaxation via a breathing method and progressive muscle relaxation, behavioral modification via activity pacing, and cognitive modification via cognitive reconstruction. The EuroQol five-dimensional questionnaire five level (EQ5D-5 L) assessment as the primary outcome and quality of life (QOL), pain severity, disability, catastrophizing, self-efficacy, and depressive symptoms as secondary outcomes were measured using self-administered questionnaires at baseline, post-treatment, and 3-month follow-up. Intention-to-treat analyses were conducted.

**Results:**

Effect size for EQ5D-5 L score was medium from baseline to post-treatment (Hedge’s *g* = − 0.72, 90% confidence interval = − 1.38 to − 0.05) and up to the 3-month follow-up (*g* = − 0.60, CI = − 1.22 to 0.02). Effect sizes for mental and role/social QOL, disability, catastrophizing, self-efficacy, and depressive symptoms were medium to large, although those for pain severity and physical QOL were small. The dropout rate was acceptably low at 14%. No severe adverse events occurred.

**Conclusion:**

The findings suggest that CBT-CP warrants a randomized controlled trial in Japan.

**Trial registration:**

University Hospital Medical Information Network Clinical Trials Registry (UMIN-CTR), UMIN000020880. Registered on 04 February 2016.

## Background

Chronic pain is pain that persists past normal healing time and lasts for > 3–6 months [1, 2]. It is related to disability, catastrophizing, and mood disorders such as depression. These conditions affect not only the patients but also their family, workplace, and society and negatively affect their quality of life (QOL) [2–4]. The prevalence of chronic pain is as high as 20% [5] and is rising every year [6]. The annual economic costs of pain associated with lower worker productivity range from $560 to $635 billion in the US [7].

Multidisciplinary chronic pain management based on bio-psycho-social models have been recommended, and cognitive behavioral therapy (CBT) is considered especially important in the treatment of chronic pain [8]. CBT addresses various problems caused by chronic pain rather than the pain per se [9, 10]; these interventions include decreasing maladaptive pain behavior and increasing adaptive behavior, identifying and modifying irrational beliefs, heightening self-efficacy to pain management, reducing psychological stress, improving physical and social function, and raising QOL. The efficacy of CBT for chronic pain (CBT-CP) has been established by many randomized controlled trials (RCT) outside Japan [11–14]. According to a systematic review [15], the effect of CBT on pain per se remains small and short term, but its effects on disability, catastrophizing, and mood are sustained over the medium and long terms. CBT-CP is also effective in improving QOL [14, 16].

Chronic pain is a serious problem in Japan as well. The prevalence of chronic pain is as high as 22.9% [17], and the annual economic costs of work loss due to chronic pain was estimated to be $19.9 billion [3]. Although CBT is recommended in Japanese treatment guidelines [18–20], manual therapy and nerve block injection are the mainstream approaches [21] and studies addressing the effect of CBT-CP in Japan have been few and insufficient. Matsubara et al. [22] conducted a study examining the effect of individual CBT among Japanese chronic pain patients, wherein treatment responders were just compared with non-responders; moreover, according to Yang et al. [23], the technical quality of therapists in that study was not well secured. Another study was a before–after trial of group CBT [24]. Group CBT has potential utility for patients through the modeling of appropriate responses from others and decreasing the patient’s feelings of being isolated and misunderstood [24], but the effect of CBT-CP itself has not yet been sufficiently verified in Japan; thus, it is a priority to establish a basic individualized CBT-CP program. This will promote the popularization of CBT-CP in Japanese clinical settings. In this context, the delay in CBT research and practice for chronic pain is a significant factor not only in Japan but also in all of Asia. It will be necessary to develop treatment methods and conduct well-designed studies to verify the effect of CBT-CP in Asia, considering cultural differences [23].

We conducted this study to develop an individualized CBT-CP program for Japanese patients, to assess its feasibility, and to inform decisions regarding sample size for future definitive trials in terms of 1) treatment efficacy with QOL as the primary outcome and pain severity, disability, psychological variables, and depression as secondary outcomes; 2) acceptability (i.e., dropout rate); and 3) safety (i.e., severe adverse events). Treatment efficacy was assessed on the basis of changes in the primary and secondary outcome measures. We expected that statistically significant changes from baseline to post-treatment and up to follow-up would be found, that the dropout rate would be < 20%, and that no severe adverse events would occur. A total of 40 studies that gave the number of participants at baseline and post-treatment assessment are referred to in a systematic review [15]. The mean dropout rate was 12.6% (standard deviation, 9.6; range, 0–34.7%). Although most of the data is for anxiety disorders, not chronic pain, the dropout rates for general CBT in controlled trials are typically 5.6–19.0% [25]. Therefore, we set a dropout rate of < 20% in this study as adequate to show the feasibility of CBT-CP.

A previous study reported that CBT-CP is effective in reducing disability, especially among patients who are significantly disabled [26]. Therefore, we also explored the hypothesis that CBT-CP would be effective in increasing QOL among patients with low QOL.

## Methods

### Trial design and ethics

The study was a pilot, open-labeled, before–after trial without a control group. We conducted the CBT-CP program among Japanese chronic pain patients and evaluated health-related QOL at baseline, post-treatment, and 3-month follow-up. The study was conducted cooperatively in five tertiary care hospitals in Japan, and the participants were recruited from each hospital. The five sites were Osaka University Hospital, Kobe University Hospital, Kawasaki Medical School Hospital, National Center of Neurology and Psychiatry Hospital, and Jikei University Hospital; ethics committees at all sites evaluated the protocols (approval numbers 15429–2; 280046; 2275–2; A2016–092; and 28–256, respectively). The study was registered in the University Hospital Medical Information Network Clinical Trials Registry (UMIN-CTR: UMIN000020880).

### Participants, consent, and permissions

Participants were outpatients who visited each site to receive medical treatment for their chronic pain. After complete explanations of the purpose and procedures of the study, we evaluated patient eligibility if they agreed to participate and signed the consent document. Patients who met the eligibility criteria were registered as participants and underwent baseline assessment. We conducted the entire eight-session CBT-CP program for all the participants. Participants underwent post-treatment assessment at 1 ± 1 week and a 3-month follow-up assessment at 12 ± 2 weeks after the last session. No incentive was given for participation.

Inclusion criteria for the study were the following: a) Chronic pain persisting for at least 3 months, b) age 20–80 years, and c) understanding of the study and informed consent based on free will. Criterion a) was set for the examination of efficacy and b) and c) were set in consideration of informed consent. Exclusion criteria were the following: a) Organic causes of pain requiring immediate medical treatment, b) alcohol or substance use disorder, c) manic episodes or diagnosis of any psychotic disorder, d) severe suicidal ideation, e) difficulty in cognitive function required to undergo CBT, f) difficulty in communication or reading and writing in Japanese, g) chronic pain due to surgery or external injury, h) compensation or lawsuit related to the chronic pain, i) pain severity 10 in a numerical rating scale (0–10), or j) unsuitability due to any other reason as judged by the attending physician. Criteria a)–f) and j) were set in consideration of safety and excluding patients who would have difficulty receiving CBT-CP continuously and stably. Criteria g) and h) were set in consideration of a potential or actual financial gain from illness. Criterion i) was set as an expert opinion of our project team that patients with 10 points tend to adhere to seeking perfect elimination of their pain and were not fit for CBT-CP.

### Outcome measures

#### EuroQol five-dimensional questionnaire five level (EQ5D-5 L)

Health-related QOL was assessed using the EQ5D-5 L [27]. We used the Japanese version of EQ5D-5 L and a Japanese scoring system that have been found to be valid and reliable [28]; a score of zero indicates death, and a score of one indicates complete health. The EQ5D-5 L score was the primary outcome.

#### Medical outcomes in the 12-item short form health survey (SF-12)

Mental and physical health-related QOL indicators were assessed using SF-12, which consists of a physical component summary (SF-12-PCS) and a mental component summary (SF-12-MCS) [29]. In Asia, including Japan, inclusion of a role/social component summary (SF-12-RCS) is recommended, and its validity has been reported [30]. The summary scores are presented as T-scores with a mean of 50 ± 10 standard deviation, with a higher score indicating a healthier status.

#### Numerical rating scale (NRS)

Pain severity was assessed using NRS with anchors of 0, indicating no pain, to 10, indicating the worst pain [31]. Participants were asked to recall the most recent week and respond with a pain score indicating 1) maximum, 2) minimum, 3) average, and 4) current. The average value of these four scores was used for analysis because it is more useful when maximal reliability is necessary [31, 32]. A higher average score indicates more severe pain.

#### Pain disability assessment scale (PDAS)

To assess disability, PDAS was used [33, 34]. PDAS measures disability in terms of capability to do physical exercise and movement among chronic pain patients. Its reliability and validity has been reported [33, 34]. A higher score indicates a higher degree of disability due to pain.

#### Patient health Questionnaire-9 (PHQ-9)

PHQ-9 is a scale for assessing mental disorders common in primary care settings per the Diagnostic and Statistical Manual of Mental Disorders [35]. We used the Japanese version of PHQ-9, for which the reliability and validity have been reported [36]. A higher score on the scale indicates lower mental health.

#### Pain Catastrophizing scale (PCS)

We used PCS to assess cognitive factors that sustain chronic pain [37, 38]. The Japanese version of PCS has been found to be valid and reliable [39]; a higher score on the scale indicates a higher degree of catastrophizing about pain.

#### Tampa scale for Kinesiophobia eleven (TSK-11)

To assess fear of movement due to musculoskeletal pain, the Japanese version of TSK-11, which has sufficient validity and reliability, was used [40]; a higher score indicates a greater fear of movement.

#### Pain self-efficacy questionnaire (PSEQ)

To assess the degree of confidence in performing a number of activities despite pain, we used PSEQ [41]. The Japanese version has been found to be valid and reliable [42]; a higher score indicates higher self-efficacy to cope with pain.

### Sample size

We based our sample size on the recommendation that ≥12 participants are suitable for pilot studies with a primary focus of estimating average values and variability to plan larger subsequent studies [43], because it was difficult to estimate sample size based on actual data due to the lack of sufficient data regarding CBT for Japanese chronic pain patients. Based on the above recommendation, 15 participants were targeted for recruitment in this study, considering dropouts.

### Interventions and clinical psychologists

The CBT-CP developed for this study is a structured and manualized eight-session program. We prepared a workbook and a worksheet with several entry columns, including one for participants to write their own examples or experiences. Each session lasted approximately 30–40 min. The program comprised three components specifically addressing chronic pain; we conducted a narrative review of eight clinical trials reported during 2009–2014 [44–51] and found that behavioral modification, cognitive modification, and relaxation were common to these trials. The outline of each session is presented in Table 1; education and information regarding the CBT model and goal setting during session 1, relaxation via a breathing method and progressive muscle relaxation during session 2, behavioral modification via activity pacing during sessions 3–5, cognitive modification via cognitive reconstruction during sessions 6–7, and summary and relapse prevention during session 8.

**Table 1:** Overview of our CBT-CP program

Session	Components
1	Education and information regarding CBT (CBT model, self-monitoring) and goal setting
2	Relaxation training (breathing method, progressive muscle relaxation)
3	Activity pacing 1 (revealing the relation between pain and behavior)
4	Activity pacing 2 (activity adjustment by limiting activities and using rest breaks)
5	Activity pacing 3 (activity adjustment by coping with obstacles)
6	Cognitive reconstruction 1 (identifying irrational beliefs related to activity adjusted in sessions 3–5, and distancing)
7	Cognitive reconstruction 2 (challenging irrational beliefs)
8	Summary and relapse prevention

*CBT-CP* Cognitive behavioral therapy for chronic pain, *CBT* Cognitive behavioral therapy

We did not use the third-generation CBT program (e.g., mindfulness-based stress reduction and acceptance and commitment therapy), because the effect sizes in the second- and third-generation CBT programs were almost equivalent at the time we developed our CBT-CP program. In addition, we surmised that the second-generation CBT would be more prevalent in Japan because there is more Japanese educational content regarding the second CBT than the third CBT program. The treatment sessions were conducted by six clinical psychologists (two with an M.A. and four with a Ph.D.) who fulfilled the following conditions: 1) ≥2 years of practice experience dealing with CBT or experience of medical examination of chronic pain patients and 2) at least one patient’s therapy fully supervised by MH and receiving approval as a CBT-CP practitioner. MH has been an expert in CBT for > 20 years. Treatment adherence was closely monitored by weekly group supervision using audiotaped recordings of all the sessions conducted in the study.

### Statistical analysis

The data were analyzed following the intent-to-treat principle. To examine the effect of CBT-CP on the primary outcome (EQ5D-5 L), a linear mixed model (LMM) was used. The LMM included a fixed effect of time and a random effect of participants. Time was treated as a categorical variable (0, baseline; 1, post-treatment; and 2, follow-up). The fixed effect of the baseline primary outcome score was also included in the LMM as covariate. The within-group standardized mean difference (Hedges’ *g*) and its 90% confidence interval were calculated using estimated marginal mean and standard error from the LMM. Secondary outcomes were analyzed using the same procedure as used for the primary outcome.

Exploratory analysis was performed that excluded participants with a primary outcome score (EQ5D-5 L) > 0.80 at baseline assessment. This analysis was conducted to estimate the effect size for populations with low QOL. No imputation for missing data was applied because the LMM can provide estimates using all the available data.

Given the preliminary and small size nature of this study, statistical significance was set at 0.10. All analyses were performed using the statistical software R version 3.5.0 and its packages [52]. LMM was performed using the lmerTest package [53], and standardized mean difference was calculated with the compute.es package [54].

## Results

### Participant flow, recruitment rate, and dropout rate

**Fig. 1:**
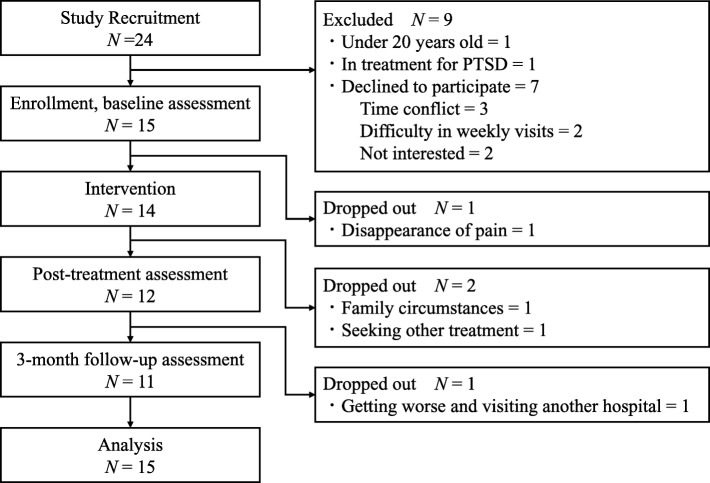
Participant flow diagram

Participants were recruited from 01 November 2016 and the last follow-up assessment was conducted on 23 January 2018. Fig. 1 depicts the participant flow. Of the 24 outpatients, 15 were eligible and were enrolled. When the number of enrollments reached the 15 participants previously targeted, we ended the recruitment. One of them dropped out because of disappearance of pain before starting session 1. Of the remaining 14, two (14%) dropped out during the intervention period. The reasons for dropout were family circumstances, after session 3, and seeking other treatment to reduce pain, after session 4. After completing all sessions of CBT-CP, one participant dropped out before follow-up assessment because of the pain getting worse and visiting another hospital. A physician in-charge from another hospital and another physician-in-attendance at the CBT-CP implementation site shared information, and they judged that the above case of exacerbation was not a side effect of CBT-CP. Age, sex, and the scores of primary outcomes (EQ5D-5 L) of the three dropout participants varied (two female and one male; ages, 56, 31, and 61 years; EQ5D-5 L scores, 0.71, 0.67, and 0.80).

### Baseline data

Table 2 presents demographic and clinical characteristics of the participants. Median pain duration was 31 (range, 6–240) months. The anatomical regions affected were the lumbar region (60%), head (47%), shoulder (40%), lower limbs (33%), and neck (33%). The number of main diagnoses described in the medical records was 12, indicating that the chronic pain types targeted in this study were mixed.

**Table 2:** Participant demographic and clinical characteristics

	Number	(%)	[Range]
Demographics	15	(100)	
Age, Mean (SD)	52.13	(14.70)	[29, 76]
Sex			
Women	9	(60)	
Men	6	(40)	
Education			
High-school or less	7	(47)	
Two year or career college graduate	3	(20)	
University graduate	4	(27)	
Master’s degree	1	(7)	
Family			
Single	3	(20)	
With partner	6	(40)	
With partner and child	2	(13)	
With Parent	3	(20)	
With partner, child, and parent	1	(7)	
Job			
Full-time worker	3	(20)	
Part-time worker	1	(7)	
Homemaker	2	(13)	
No job (Older age)	2	(13)	
No job (Cause of pain)	4	(27)	
No job (Cause other than pain)	2	(13)	
Other	1	(7)	
Pain-related characteristics			
Duration (Month), Median	31		[6, 240]
Region of pain (multiple answers allowed)			
Lumbar	9	(60)	
Head/Face/Mouth	7	(47)	
Shoulder/Upper limbs	6	(40)	
Lower limbs	5	(33)	
Neck	5	(33)	
Abdomen	2	(13)	
Chest	2	(13)	
Genital/Anus/Perineum	2	(13)	
Pelvis	1	(7)	
Main diagnosis described in a medical record			
Chronic primary pain	3	(20)	
Fibromyalgia	2	(13)	
Adhesive capsulitis	1	(7)	
Annal pain	1	(7)	
Atypical facial neuralgia	1	(7)	
Atypical odontalgia	1	(7)	
Cubital tunnel syndrome	1	(7)	
Glossodynia	1	(7)	
Intercostal neuralgia	1	(7)	
Occipital neuralgia	1	(7)	
Ossification of posterior longitudinal ligament	1	(7)	
Sciatica neuralgia	1	(7)	

### Outcome measures

Table 3 presents the mean and standard deviation of the primary and secondary outcome measures at baseline, post-treatment, and follow-up assessment. Estimated mean differences (*MD*) and standardized mean differences (Hedges’ *g*) of the outcome measures with 90% confidence interval are shown in Table 4.

**Table 3:** Descriptive statistics of outcome measures (mean and standardized deviation)

	Baseline (*N* = 15)		Post-treatment (*N* = 12)		Follow-up (*N* = 11)	
	Mean	SD	Mean	SD	Mean	SD
Over all QOL measures						
EQ5D-5 L (primary outcome)	0.66	0.15	0.75	0.18	0.75	0.15
SF-12-PCS	32.20	15.97	32.64	14.56	37.96	14.18
SF-12-MCS	44.89	10.70	53.00	9.74	49.96	9.39
SF-12-RCS	39.61	12.94	47.60	13.12	47.49	13.20
Pain severity/Disability						
NRS summary score	3.70	1.23	3.65	1.09	4.03	0.86
PDAS	18.47	10.82	15.25	9.73	16.73	14.39
Pain-related psychological variables						
PCS	30.53	11.58	19.25	11.29	17.64	10.28
TSK-11	26.00	5.55	23.42	4.85	22.36	4.78
PSEQ	28.87	12.34	38.33	12.96	41.18	8.80
Depressive symptom						
PHQ-9 total	8.73	6.09	5.25	3.91	4.64	4.30

*QOL* Quality of life, *EQ5D-5 L* EuroQol five-dimensional questionnaire five level, *SF-12-PCS* Medical Outcomes Study 12-Item Short Form Health Survey-Physical Component Summary, *MCS* Mental Component Summary, *RCS* Role/Social Component Summary, *NRS* Numerical Rating Scale, *PDAS* Pain Disability Assessment Scale, *PCS* Pain Catastrophizing Scale, *TSK-11* Tampa Scale for Kinesiophobia eleven, *PSEQ* Pain Self-Efficacy Questionnaire, *PHQ-9* Patient Health Questionnaire-9

**Table 4:** Estimated mean difference and standardized mean difference with 90% confidence interval from LMM

	Baseline to post-treatment		Baseline to follow-up	
	MD	SMD (Hedge’s *g*)	MD	SMD(Hedge’s *g*)
Overall QOL measures				
EQ5D-5 L (primary outcome)	**0.10 [0.03, 0.18]**	**−0.72 [−1.38, − 0.05]**	**0.09 [0.01, 0.16]**	**−0.60 [−1.22, 0.02]**
SF-12-PCS	1.78 [−4.06, 7.63]	−0.18 [− 0.82, 0.47]	5.49 [− 0.50, 11.48]	− 0.53 [− 1.15, 0.08]
SF-12-MCS	**7.70 [2.12, 13.28]**	**−0.80 [−1.46, − 0.13]**	5.45 [− 0.27, 11.17]	− 0.55 [− 1.17, 0.06]
SF-12-RCS	**8.21 [3.56, 12.87]**	**−0.94 [−1.62, − 0.26]**	**7.10 [2.32, 11.89]**	**−0.80 [−1.43, − 0.17]**
Pain severity / Disability				
NRS summary score	−0.21 [− 0.83, 0.40]	0.19 [− 0.45, 0.84]	0.19 [− 0.44, 0.82]	− 0.17 [− 0.78, 0.44]
PDAS	−4.85 [−10.33, 0.63]	0.51 [−0.14, 1.16]	−2.34 [−7.94, 3.26]	0.24 [−0.36, 0.85]
Pain-related psychological variables				
PCS	**−11.04 [− 17.23, −4.85]**	**0.98 [0.30, 1.67]**	**−12.34 [− 18.69, −5.98]**	**1.08 [0.43, 1.73]**
TSK-11	−2.83 [−6.03, 0.37]	0.51 [−0.14, 1.16]	**−3.91 [− 7.19, − 0.63]**	**0.70 [0.07, 1.32]**
PSEQ	**9.89 [3.66, 16.12]**	**−0.85 [−1.52, − 0.18]**	**12.21 [5.81, 18.61]**	**−1.03 [− 1.68, − 0.39]**
Depressive symptoms				
PHQ-9 total	**−3.45 [−5.75, −1.14]**	**0.86 [0.19, 1.54]**	**−3.95 [−6.31, −1.58]**	**0.97 [0.33, 1.62]**

*LMM* Linear mixed model, *MD* Mean difference, *SMD* Standardized mean difference (Hedge’s g), *QOL* Quality of life, *EQ5D-5 L* EuroQol five-dimensional questionnaire five level, *SF-12-PCS* Medical Outcomes Study 12-Item Short Form Health Survey-Physical Component Summary, *MCS* Mental Component Summary, *RCS* Role/Social Component Summary, *NRS* Numerical Rating Scale, *PDAS* Pain Disability Assessment Scale, *PCS* Pain Catastrophizing Scale, *TSK-11* Tampa Scale for Kinesiophobia eleven, *PSEQ* Pain Self-Efficacy Questionnaire, *PHQ-9* Patient Health Questionnaire-9

#### Primary outcome measures

The primary outcome (EQ5D-5 L) improved significantly from baseline to post-treatment (*MD* = 0.10 [0.03, 0.18]) and follow-up (*MD* = 0.09 [0.01, 0.16]). The effect size of the EQ5D-5 L was medium from baseline to post-treatment (*g* = − 0.72 [− 1.38, − 0.05]) and follow-up (*g* = − 0.60 [− 1.22, 0.02]). The confidence intervals were wide.

#### Secondary outcome measures

Regarding secondary QOL measures, significant improvement was observed in the SF-12-MCS and SF-12-RCS from baseline to post-treatment (*g* = − 0.80 [− 1.46, − 0.13]; *g* = − 0.94 [− 1.62, − 0.26]). Between baseline and follow-up, improvement in the SF-12-RCS was statistically significant (*g* = − 0.80 [− 1.43, − 0.17]), but that of the SF-12-MCS was not (*g* = − 0.55 [− 1.17, 0.06]). The change in the SF-12-PCS was not significant from baseline to post-treatment (*g* = − 0.18 [− 0.82, 0.47]) or follow-up (*g* = − 0.53 [− 1.15, 0.08]).

Regarding pain severity and disability, the NRS summary score and PDAS did not improve significantly from baseline to post-treatment (*g* = 0.19 [− 0.45, 0.84]; *g* = 0.51 [− 0.14, 1.16]) or follow-up (*g* = − 0.17 [− 0.78, 0.44], *g* = 0.24 [− 0.36, 0.85]).

Regarding pain-related psychological variables, PCS and PSEQ improved significantly from baseline to post-treatment (*g* = 0.98 [0.30, 1.67]; *g* = − 0.85 [− 1.52, − 0.18]) and follow-up (*g* = 1.08 [0.43, 1.73], *g* = − 1.03 [− 1.68, − 0.39]). The TSK-11 did not improve significantly from baseline to post-treatment (*g* = 0.51 [− 0.14, 1.16]) but showed improvement from baseline to follow-up (*g* = 0.70 [0.07, 1.32]).

Regarding depressive symptoms, the PHQ-9 improved significantly from baseline to post-treatment (*g* = 0.86 [0.19, 1.54]) and follow-up (*g* = 0.97 [0.33, 1.62]).

### Adverse events

No severe adverse events related to study participation occurred during the intervention period, but adverse events were experienced by two participants (family conflict by one and onset of physical illness (endometriosis) by the other). These adverse events were rated as having a non-causal relationship with study participation.

### Exploratory analysis

Table 5 presents estimated and standardized mean differences of the primary and secondary outcomes among 12 participants whose EQ5D-5 L scores were ≤ 0.80. EQ5D-5 L improved significantly from baseline to post-treatment (MD = 0.18 [0.09, 0.27]) and follow-up (MD = 0.15 [0.06, 0.24]). The effect size of the EQ5D-5 L was larger than the entire sample from baseline to post-treatment (*g* = − 1.24 [− 2.14, − 0.34]) and follow-up (*g* = − 1.01 [− 1.88, − 0.14]). A similar trend was observed in the secondary outcomes.

**Table 5:** Estimated mean difference and standardized mean difference in low QOL participants (EQ5D-5 L ≤0.80)

	Baseline to post-treatment		Baseline to follow-up	
	MD	SMD (Hedge’s *g*)	MD	SMD(Hedge’s *g*)
Overall QOL measures				
EQ5D-5 L (primary outcome)	**0.18 [0.09, 0.27]**	**−1.24 [−2.14, −0.34]**	**0.15 [0.06, 0.24]**	**−1.01 [− 1.88, − 0.14]**
SF-12-PCS	3.97 [−4.62, 12.56]	−0.32 [−1.14, 0.50]	7.51 [−1.35, 16.36]	−0.59 [−1.42, 0.25]
SF-12-MCS	**11.01 [4.20, 17.83]**	**−1.12 [−2.00, −0.23]**	7.84 [0.74, 14.95]	−0.76 [−1.61, 0.08]
SF-12-RCS	**12.11 [6.21, 18.02]**	**−1.33 [−2.24, −0.42]**	**8.85 [2.71, 14.98]**	**−0.94 [−1.80, − 0.07]**
Pain severity / Disability				
NRS summary score	**−1.00 [− 1.66, −0.34]**	**0.96 [0.09, 1.83]**	−0.48 [−1.17, 0.20]	0.45 [−0.38, 1.28]
PDAS	−8.00 [−15.86, −0.14]	0.71 [−0.14, 1.55]	−3.75 [−11.83, 4.33]	0.32 [−0.50, 1.14]
Pain-related psychological variables				
PCS	**−15.99 [−23.54, −8.43]**	**1.40 [0.48, 2.32]**	**−15.76 [−23.6, −7.93]**	**1.34 [0.43, 2.25]**
TSK-11	−3.81 [−8.37, 0.75]	0.58 [−0.26, 1.41]	−4.23 [−8.96, 0.50]	0.62 [−0.22, 1.46]
PSEQ	**14.14 [5.90, 22.38]**	**−1.12 [−2.00, −0.24]**	**16.03 [7.48, 24.57]**	**−1.23 [−2.12, −0.33]**
Depressive symptoms				
PHQ-9 total	**−5.56 [−8.46, −2.67]**	**1.33 [0.42, 2.24]**	**−4.82 [−7.82, −1.83]**	**1.11 [0.23, 1.99]**

*MD* Mean difference, *SMD* Standardized mean difference (Hedge’s *g*), *QOL* Quality of life, *EQ5D-5 L* EuroQol five-dimensional questionnaire five level, *SF-12-PCS* Medical Outcomes Study 12-Item Short Form Health Survey-Physical Component Summary, *MCS* Mental Component Summary, *RCS* Role/Social Component Summary, *NRS* Numerical Rating Scale, *PDAS* Pain Disability Assessment Scale, *PCS* Pain Catastrophizing Scale, *TSK-11* Tampa Scale for Kinesiophobia eleven, *PSEQ* Pain Self-Efficacy Questionnaire, *PHQ-9* Patient Health Questionnaire-9

## Discussion

This pilot study suggests that CBT-CP is feasible for the treatment of chronic pain patients in Japan. CBT-CP promoted statistically significant changes in terms of the primary outcome and some secondary outcomes, with medium-to-large effect sizes. The dropout rate was acceptably low, and no severe adverse events occurred. Most results were in agreement with the hypothesis and indicated that CBT-CP is feasible for use in the Japanese population.

### Potential efficacy in terms of QOL, pain severity, disability, psychological variables, and depressive symptoms

CBT-CP demonstrated medium-to-large effect sizes in terms of QOL. The effect size of EQ5D-5 L as a primary outcome was medium, both post-treatment and at follow-up. Regarding secondary outcomes, the effect sizes were large for SF-12-MCS post-treatment and for SF-12-RCS at both post-treatment and follow-up. Therefore, mental and role/social health-related QOL improved significantly, and in general, health-related QOL improved with a greater than medium effect size, suggesting that CBT-CP has potential efficacy among Japanese chronic pain patients in terms of QOL improvement. Further, exploratory analysis revealed that the effect size was larger among patients whose QOL was ≤0.80, both post-treatment and at follow-up. This suggests that patients with low QOL receive more benefit from CBT-CP, as also seen in terms of disability [26].

Although the effect of CBT-CP on pain per se was not assessed, it had a small effect on disability in daily life caused by pain. As shown in a systematic review [15], the effect size for NRS was insignificant and small, both post-treatment and at follow-up. On the other hand, although not significant, the effect size was medium for PDAS post-treatment and for SF-12-PCS at follow-up. A systematic review showed that CBT has a small effect on disability, both in the short and long term [15]. This suggests that CBT-CP is not effective against pain per se, but is potentially effective against disability caused by pain in the Japanese population as well.

CBT-CP contributed significantly to improvement in psychological variables and depressive symptoms related to pain. In a systematic review, CBT has been shown to have a medium effect on catastrophizing in the short term and a small effect in the long term [15]. In this study, CBT-CP had a large effect size on catastrophizing, a medium effect on fear of movement, and a large effect on self-efficacy in managing pain. CBT-CP did not reduce pain per se, but decreased catastrophizing and fear of movement due to pain, thereby empowering patients to manage pain themselves. CBT has been shown to have a small-to-medium effect, both in the short term and the long term, in a systematic review [15]; consistent with this, depressive symptoms were significantly improved both post-treatment and at follow-up in this study. In light of the fear-avoidance model [55], CBT-CP improved catastrophizing, fear of movement, and inactivity, which might reduce depressive symptoms. Thus, CBT may be potentially effective among Japanese chronic pain patients in terms of modifying cognition and attitude toward pain and improving the emotional state.

### Potential acceptability and safety of CBT-CP

The dropout rate was 14%, and there were no severe events that occurred as side effects of CBT-CP during the intervention period. This suggests that the CBT-CP program developed in this study has sufficient acceptability and safety.

### Limitations

There are two limitations of this study. First, it was a before–after trial without a control group. Regarding effect size, although some effect sizes shown in the study were larger than those shown in a systematic review based on RCTs [15], the effect sizes in before–after trials tend to be larger than those of RCTs. Regarding the durability of effect, although there were > 50% of the variables with medium-to-large effect sizes over a 3-month follow-up, we do not have sufficient data to address the causes. Therefore, future definitive RCTs need to be conducted that address the extent and duration of efficacy of CBT-CP among Japanese chronic pain patients.

Second, the chronic pain types targeted in this study were mixed. Globally, it is important to examine the effect of CBT-CP on each diagnosis or disease condition constituting chronic pain because there is sufficient evidence that CBT-CP is effective in treating chronic mixed pain [15]. For example, some studies have indicated CBT-CP is effective for chronic low back pain [12, 56, 57], but not for chronic neck pain [58]. Although differences in cultural background may also have influenced the characteristics of chronic pain and the effect of CBT-CP, studies examining this aspect are insufficient, especially in Asia, including Japan [23]. Therefore, it will be important to conduct more studies on individual chronic pain in Asia following adequate studies on chronic mixed pain.

## Conclusions

CBT is potentially effective for improving QOL and various psychological variables among Japanese chronic pain patients, and the CBT-CP program developed for this study is feasible for use among the Japanese because of low the dropout rate and no severe adverse events related to study participation. Further, development of structured and manualized CBT-CP programs with workbooks and worksheets will contribute not only to future studies but also to clinical practice in Japan. Future definitive RCTs with a control group are needed to carefully examine the effect of CBT among Japanese chronic pain patients, followed by clinical trials addressing each diagnosis and disease condition constituting chronic pain in Asia, including Japan.

## Data Availability

The datasets used and/or analyzed during the current study are available from the corresponding author on reasonable request.

## Abbreviations

CBT: Cognitive behavioral therapy
CBT-CP: CBT for chronic pain
QOL: Quality of life
RCT: Randomized controlled trials
UMIN-CTR: University hospital medical information network clinical trials registry
EQ5D-5 L: EuroQol five-dimensional questionnaire five level
SF-12: Medical outcomes in the 12-item short form health survey.
SF-12-PCS: SF-12 physical component summary.
SF-12-MCS: SF-12 mental component summary.
SF-12-RCS: SF-12 role/social component summary.
NRS: Numerical rating scale
PDAS: Pain disability assessment scale
PHQ-9: Patient health questionnaire-9
PCS: Pain catastrophizing scale
TSK-11: Tampa scale for kinesiophobia eleven
PSEQ: Pain self-efficacy questionnaire
LMM: Linear mixed model
MD: Mean differences
